# Phosphorylation of Not4p Functions Parallel to *BUR2* to Regulate Resistance to Cellular Stresses in *Saccharomyces cerevisiae*

**DOI:** 10.1371/journal.pone.0009864

**Published:** 2010-04-08

**Authors:** Nga-Chi Lau, Klaas W. Mulder, Arjan B. Brenkman, Shabaz Mohammed, Niels J. F. van den Broek, Albert J. R. Heck, H. Th. Marc Timmers

**Affiliations:** 1 Department of Physiological Chemistry, University Medical Center Utrecht, Utrecht, The Netherlands; 2 Netherlands Proteomics Centre, Utrecht, The Netherlands; 3 Biomolecular Mass Spectrometry and Proteomics Group, Bijvoet Center for Biomolecular Research, Utrecht University, Utrecht, The Netherlands; Texas A&M University, United States of America

## Abstract

**Background:**

The evolutionarily conserved Ccr4-Not and Bur1/2 kinase complexes are functionally related in *Saccharomyces cerevisiae*. In this study, we further explore the relationship between the subunits Not4p and Bur2p.

**Methodology/Principal Findings:**

First, we investigated the presence of post-translational modifications on the Ccr4-Not complex. Using mass spectrometry analyses we identified several SP/TP phosphorylation sites on its Not4p, Not1p and Caf1p subunits. Secondly, the influence of Not4p phosphorylation on global H3K4 tri-methylation status was examined by immunoblotting. This histone mark is severely diminished in the absence of Not4p or of Bur2p, but did not require the five identified Not4p phosphorylation sites. Thirdly, we found that Not4p phosphorylation is not affected by the kinase-defective *bur1-23* mutant. Finally, phenotypic analyses of the Not4p phosphomutant (*not4S/T5A*) and *bur2Δ* strains showed overlapping sensitivities to drugs that abolish cellular stress responses. The double-mutant *not4S/T5A* and *bur2Δ* strain even revealed enhanced phenotypes, indicating that phosphorylation of Not4p and *BUR2* are active in parallel pathways for drug tolerance.

**Conclusions:**

Not4p is a phospho-protein with five identified phosphorylation sites that are likely targets of a cyclin-dependent kinase(s) other than the Bur1/2p complex. Not4p phosphorylation on the five Not4 S/T sites is not required for global H3K4 tri-methylation. In contrast, Not4p phosphorylation is involved in tolerance to cellular stresses and acts in pathways parallel to *BUR2* to affect stress responses in *Saccharomyces cerevisiae*.

## Introduction

The evolutionarily conserved Ccr4-Not complex consists of nine core subunits in *Saccharomyces cerevisiae* and regulates mRNA biogenesis at multiple levels (reviewed in [1], [2]). The Ccr4-Not complex can both negatively and positively regulate gene transcription [3], [4], and its Ccr4p and Caf1p subunits initiate mRNA degradation by their cytoplasmic deadenylase activity [5]. Beside this enzymatic activity, a protein ubiquitin ligase (E3) function has been described for the RING domain of Not4p [6]. The interaction of Not4p with ubiquitin-conjugating enzymes (E2s) Ubc4p and Ubc5p is required for a proper stress response to drugs like hydroxyurea and hygromycin B [6]. Moreover, Not1p, Not3p, Not5p and Caf1p are phosphoproteins that probably play a role in the signal transduction cascade in stress responses [1].

Synthetic lethal interactions of several *CCR4-NOT* genes with *BUR1* and *BUR2* have been observed [7]. The *BUR* genes have been identified in a genetic screen for mutations that increase transcription from the basal *SUC2* promoter in yeast [8]. *BUR2* encodes a cyclin for the essential cyclin-dependent protein kinase (CDK) Bur1p [9]. This Bur1/2p CDK/cyclin-pair is involved in transcription elongation [10], [11], [12] and activates polymerase II promoters by facilitating histone H3 lysine-4 tri-methylation (H3K4me3) [13], [14], [15]. H3K4 tri-methylation is mediated by the Set1p-complex/COMPASS complex in yeast, which requires ubiquitination of histone H2B and Bur1/2p-facilitated PAF complex recruitment [13], [14]. Notably, Not4p and other Ccr4-Not subunits are also required for the H3K4me3 mark [7], [16], [17]. It has been suggested that the H3K4-specific demethylase Jhd2p is a direct substrate for the E3 ligase activity of Not4p [17], but E3 ligase-inactive Not4p mutants did not display reduced levels of H3K4 methylation [7]. It remains unclear how Ccr4-Not subunits function in relation to the Bur1/2p kinase complex.

To investigate the functional relationship between the Ccr4-Not and Bur1/2 kinase complexes, we first explore the phosphorylation status of Ccr4-Not components. We confirmed that Not4p is a phospho-protein *in vivo*. Mass spectrometry analyses identified several serine/threonine sites as phosphoacceptor-sites for Not4p. Substitution of these sites to alanine (Not4p-S/T5A) indicates that the mechanism by which the Ccr4-Not complex and the Bur1/2p complex regulate H3K4 tri-methylation is independent of Not4p phosphorylation. In addition, we found that a severe kinase-defective allele of *BUR1* did not affect Not4p phosphorylation. Further analysis indicates that Not4p phosphorylation is functionally important for tolerance to drugs that induce replication stress and protein translation errors. Yeast strains containing the Not4p penta-phosphomutant in combination with a *BUR2* deletion show a more severe phenotype than either single mutant, which argues against a linear pathway relationship between *NOT4* and *BUR2*. Taken together, our data indicate that phosphorylation of Not4p is involved in tolerance of DNA replication stress and protein processing errors, likely in pathways parallel to *BUR2*.

## Results

### Not4p is a phospho-protein *in vivo*

**Figure 1:**
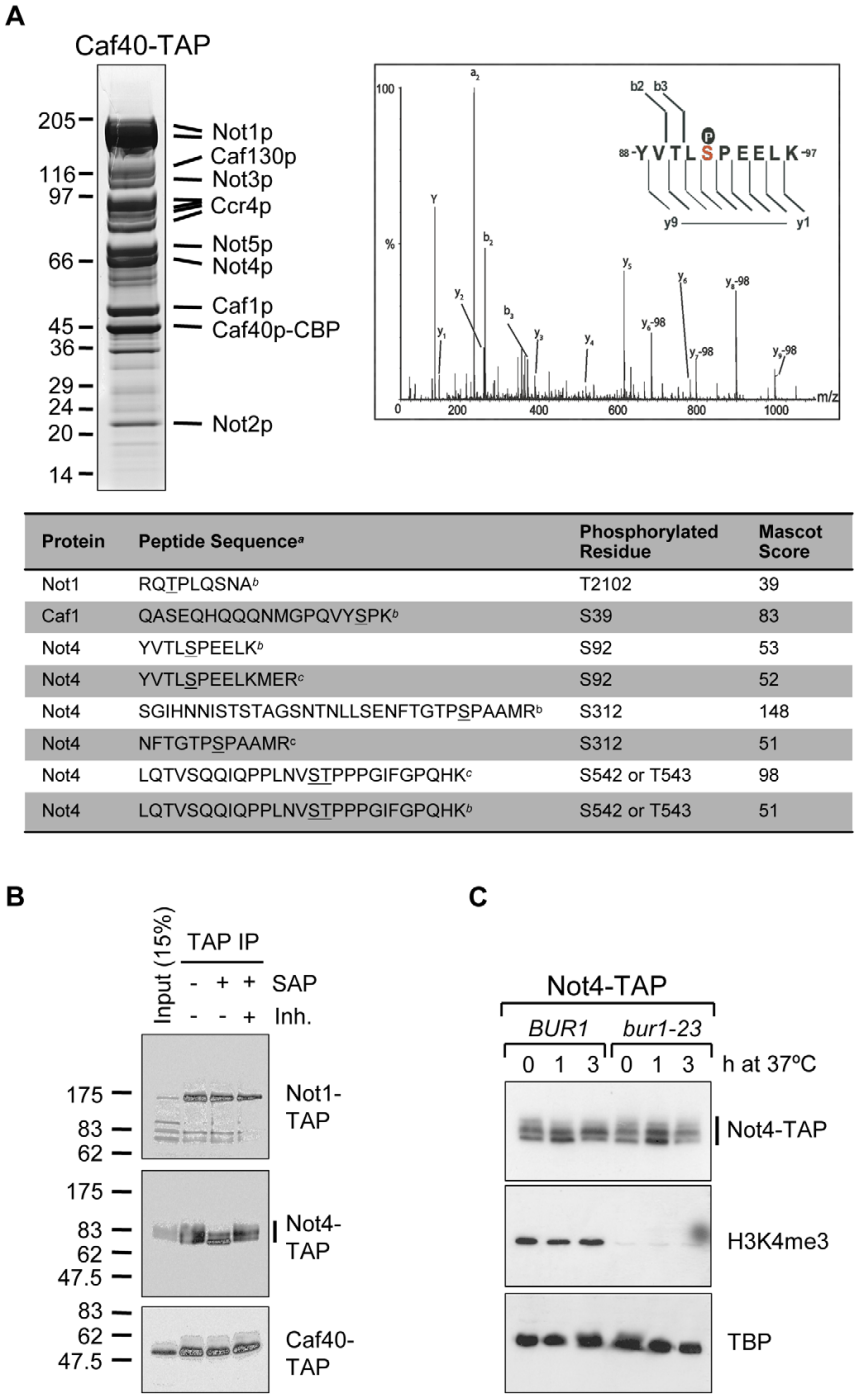
Not4p is a phosphoprotein. **A:** Phospho-proteomics on the Ccr4-Not complex. Ccr4-Not complexes were TAP-tagged purified from a strain expressing Caf40-TAP and visualized on gradient SDS-PAGE gel by Coomassie (upper left panel), marker proteins (kDa) are indicated on the left. Tryptic digestion of Coomassie stained bands was followed by LC-MS/MS analyses, leading to the identification of the Ccr4-Not subunits and their phosphorylated peptides (inserted Table; *^a^* Phosphorylated amino acids are underlined; *^b^* Cleaved with trypsin and detected by ESI-QTOF mass spectrometry; *^c^* Cleaved with trypsin/V8 and detected by ESI-LTQ-Orbitrap mass spectrometry). A representative spectrum including peak assignment of Not4p phosphorylation on S92 is given (upper right panel; inset represents the b- and y-ion coverage of the phosphopeptide). **B:** Not4p is phosphorylated *in vivo*. TAP-tagged versions of Not1p, Not4p or Caf40p were captured on IgG beads and subjected to treatment with shrimp alkaline phosphatase (SAP) or SAP pre-incubated with phosphatase inhibitors (Inh.). Samples were resolved by SDS-PAGE and analyzed by immunoblotting using antibodies recognizing the protein A moiety of the TAP-tag (anti-PAP). Marker proteins (kDa) are indicated on the left. **C:** Bur1p kinase activity is not required for phosphorylation of Not4p. Strains expressing Not4-TAP and either the *BUR1* or the *bur1-23* allele were incubated at 37°C for the indicated hours (h). Samples were analyzed by immunoblotting with antibodies against PAP, H3K4me3 or TBP.

The shared function of the Ccr4-Not and the Bur1/2 kinase complexes in H3K4 tri-methylation [7] prompted us to test involvement of this kinase complex in post-translational modification of the Ccr4-Not complex. To identify the phosphorylation sites on its subunits, mass spectrometry analyses were performed on the purified Ccr4-Not complex using Caf40-TAP as the bait (Figure 1A). Ccr4-Not components were in-gel digested with trypsin or trypsin/V8 and subjected to LC-MS/MS analyses. Unique phospho-peptides, corresponding to Not1p (T2102), Caf1p (S39) and Not4p (S92, S312, and S542 or T543), were identified (Figure 1A). These sites correspond to phospho-sites identified in large-scale phospho-proteome analyses [18], [19], [20]. Notably, Not1p, Caf1p and Not4p were phosphorylated on SP and TP sites, which are often targets of CDK/cyclin-pair kinases. In addition, another Not4p peptide (318–361 AQLHHDSHTNAGTPVLTPAPVPAGSNPWGVTQSATPVTSINLSK) was identified by mass spectrometry as a singly-phosphorylated peptide (data not shown), but the phospho-acceptor site could not be identified unequivocally. Manual inspection of the MS/MS spectrograms indicates that T334 and/or S342 of this Not4p peptide are the most likely phospho-acceptor sites. The phosphorylation status of several Ccr4-Not subunits was assessed by their electrophoretic mobility upon dephosphorylation (Figure 1B). TAP-tagged proteins (Not1p, Not4p or Caf40p) were captured on IgG-Sepharose beads and subjected to treatment with shrimp alkaline phosphatase (SAP). As a control, SAP was inactivated using phosphatase inhibitors prior to the reaction. Interestingly, the phosphatase-activity dependent mobility was increased for Not4p, but not for Not1p or Caf40p. This confirms that Not4p is a phosphorylated protein. To further investigate the involvement of the Bur1/2p kinase complex in Not4p phosphorylation, electrophoretic migration of Not4p was assessed from Not4p-TAP yeast containing the wild-type *BUR1* or the temperature-sensitive *bur1-23* allele. The latter yeast strain possesses severely decreased kinase activity, even at permissive temperature [10]. Clearly, the electrophoretic mobility of Not4p was not affected in the *bur1-23* yeast, whereas the level of H3K4 tri-methylation has significantly decreased as expected (Figure 1C). This suggests that efficient phosphorylation of Not4p is not dependent on Bur1p kinase activity.

### The Not4p penta-phosphomutant displays wild-type levels of H3K4me3

**Figure 2:**
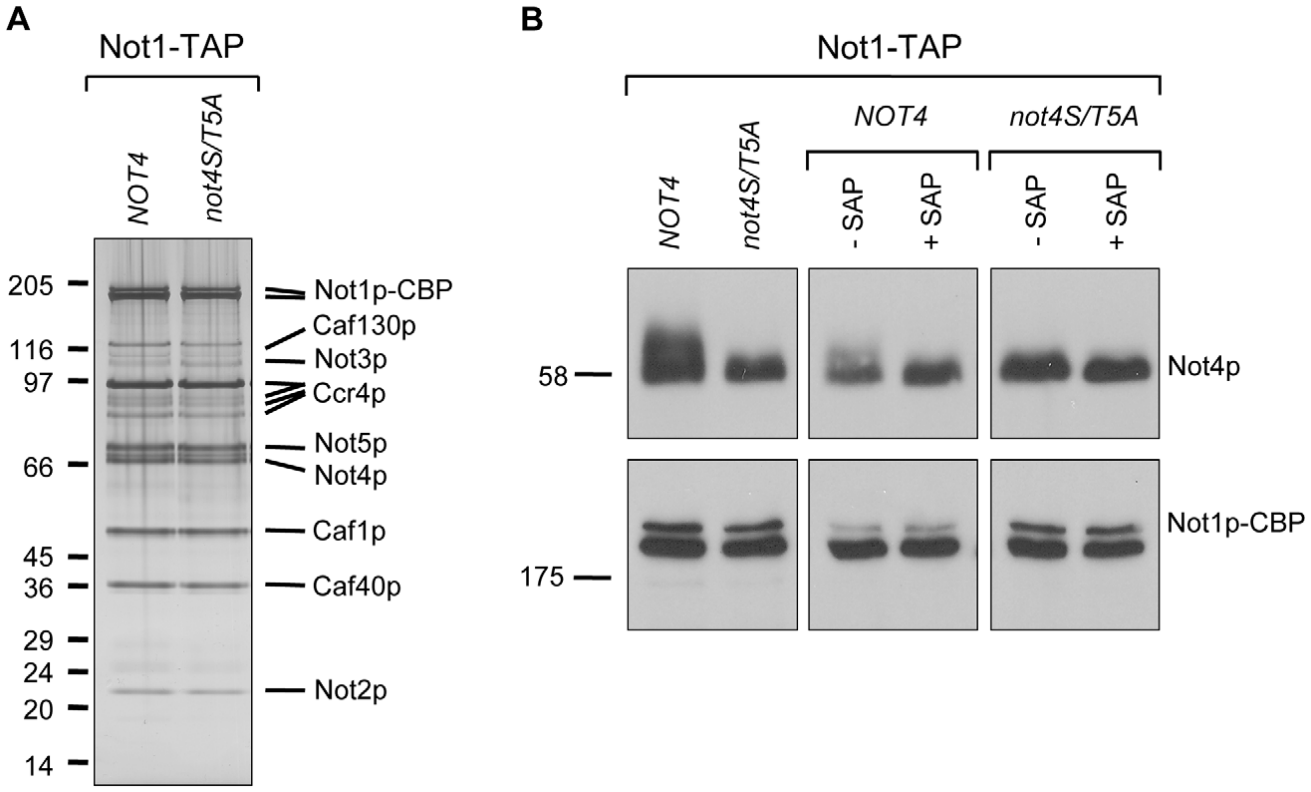
The penta-phosphomutant of Not4p has increased electrophoretic mobility. **A**: The identified phospho-sites of Not4p are not required for Ccr4-Not complex assembly. Ccr4-Not complexes were TAP-tagged purified from a strain expressing Not1-TAP containing or lacking the Not4p penta-phosphomutant (*not4S/T5A*). Purified proteins were visualized by silver staining on a gradient SDS-PAGE gel. Marker proteins (kDa) are indicated on the left. **B:** Mutation of the five phospho-sites of Not4p results in increased gel migration. Purified proteins from panel **A** were subjected to (mock-) SAP treatment and analyzed by immunoblotting with antibodies against Not1p or Not4p. Marker proteins (kDa) are indicated on the left.

To further investigate the effect of Not4p phosphorylation, phospho-Not4p mutants were generated by substitution of the identified SP/TP phospho-sites and putative phospho-sites on Not4p to alanine (*not4S/T5A*). To this end, wild-type *NOT4* or the *not4S/T5A* mutant allele was chromosomally integrated into the NCY1 strain, which carries a TAP-tagged *NOT1* allele and a *NOT4* deletion (see also Table 1). Ccr4-Not complexes were isolated from these yeast strains using Not1-TAP as the bait. Mutation of Not4p phosphorylation-sites did not affect the assembly of the Ccr4-Not core complex (Figure 2A). Furthermore, purified Ccr4-Not complexes from both *NOT4* as *not4S/T5A* strains were subjected to electrophoretic migration analyses. Notably, Not4p-S/T5A shows an increased migration compared to wild-type Not4p (Figure 2B, upper left panel), that did not change upon SAP treatment (Figure 2B, right upper panel). As expected, mobility of wild-type Not4p was increased by SAP activity (Figure 2B, middle upper panel). This confirms that the identified Not4p phosphorylation sites are responsible for the observed electrophoretic mobility change.

**Table 1:** *Saccharomyces cerevisiae* strains used in this study.

Strain	Genotype	Source
BY4741	MATa *his3Δ1 leu2Δ0 met15Δ0 ura3Δ0*	EUROSCARF
KMY58	Isogenic to BY4741 except *not4:KanMX6*	EUROSCARF
KMY161	Isogenic to BY4741 except *bur2:KanMX6*	EUROSCARF
DS1	MATa *ade2 arg4(RV-) leu2-3112 trp1-289 ura3-52*	[21]
KMY90	Isogenic to DS1 except *set1:URA3*	[21]
KMY164	Isogenic to DS1 except *CAF40-TAP:URA3*	This work
KMY86	Isogenic to BY4741 except *NOT1-TAP:URA3*	This work
KMY87	Isogenic to BY4741 except *NOT4-TAP:URA3*	This work
KMY88	Isogenic to BY4741 except *CAF40-TAP:URA3*	This work
YSB787	MATa *bur1:HIS3 ura3-52 leu2Δ1 trp1Δ63 his3Δ200 lys2Δ202 (pRS316-BUR1)*	[10]
KMY143	Isogenic to YSB787 except *NOT4-TAP:TRP1 (pRS315-BUR1 HA3)* a	This work
KMY145	Isogenic to YSB787 except *NOT4-TAP:TRP1 (pRS315-bur1-23 HA3)* a	This work
NCY1	Isogenic to KMY86 except *not4:KanMX6*	This work
NCY2	Isogenic to NCY1 except *NOT4:LEU2*	This work
NCY16	Isogenic to NCY1 except *not4S/T5A:LEU2* b	This work
NCY29	Isogenic to KMY58 except *not4S/T5A:LEU2* b	This work
NCY35	Isogenic to KMY58 except *NOT4:LEU2*	This work
2922	MATα *mfa1Δ::MFA1pr-HIS3 his3Δ1 ura3Δ0 lys2Δ0 can1Δ*	[28]
KMY187	Isogenic to 2922 except *bur2:URA3*	This work
NCY37	Isogenic to NCY29 except *bur2:URA3*	This work
NCY43	Isogenic to NCY35 except *bur2:URA3*	This work
NCY45	Isogenic to BY4741 except *bur2:URA3*	This work

*^a^*subjected to 5-FOA selection.

*^b^not4S/T5A  =  not4S92A/S312A/T334A/S342A/T543A.*

**Figure 3:**
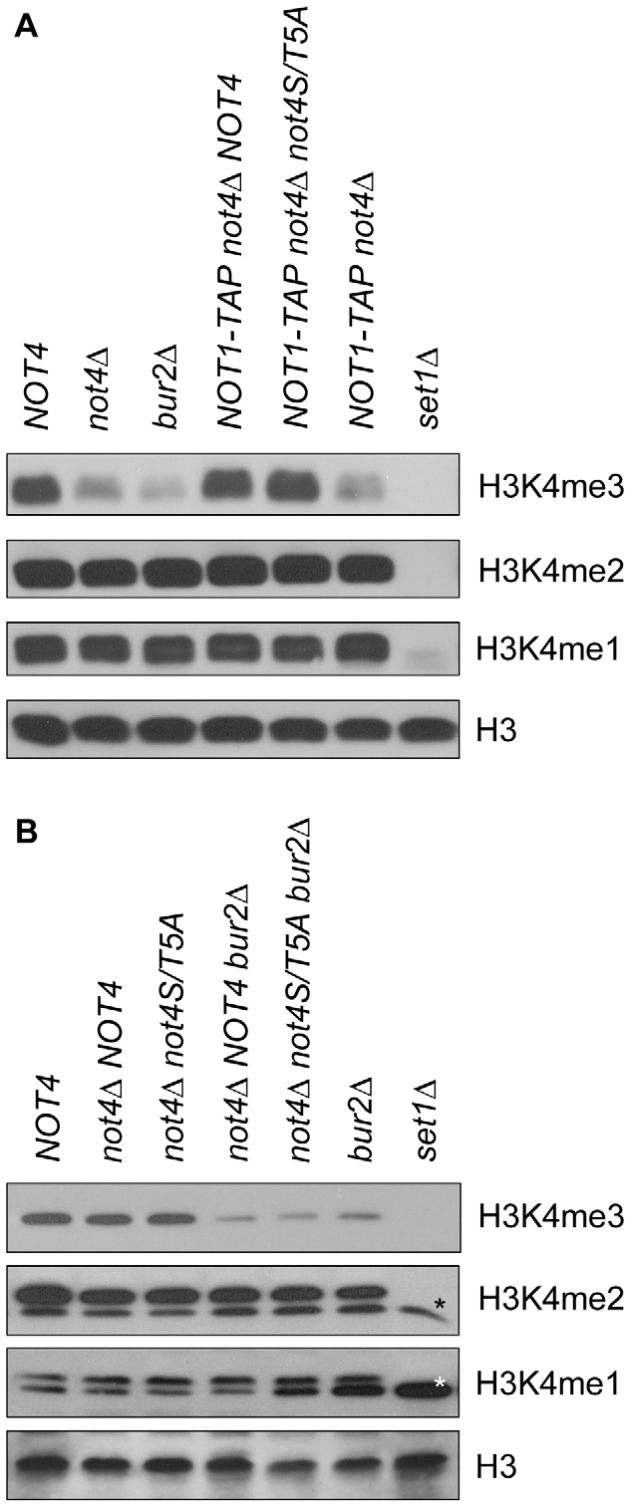
Not4p phosphorylation has no influence on global H3K4 tri-methylation. Extracts of strains indicated on the top were subjected to immunoblotting using the indicated antibodies on the right. The *SET1* deletion strain was used as an antibody specificity control. **A**: Mutation of all five phospho-sites of Not4p does not affect H3K4 tri-methylation. **B**: Addition of the Not4p penta-phosphomutant (*not4S/T5A*) into the *bur2Δ* strain does not enhance its H3K4 tri-methylation defect. Asterisks indicate aspecific bands.

Not4p and Bur2p were reported previously to be involved in the regulation of H3K4 tri-methylation [7], [13], [14], [16], [17]. We examined whether the phospho-sites of Not4p are required for this methylation event. Cellular extracts of yeast containing the penta-phosphomutant of Not4p (*not4S/T5A*) were analysed by immunoblot analysis (Figure 3). In agreement with previous observations [7], [13], [14], [16], [17], deletion of *NOT4* or *BUR2* resulted in severely decreased levels of H3K4me3 (Figure 3A). The *SET1* deletion strain serves as a control for abolished tri-, di-, and mono-methylation levels of H3K4 (Figure 3) [21], [22]. In contrast, the *not4S/T5A* strain shows wild-type levels of H3K4me3 (Figure 3). No additional effects on tri-methylation levels by the *not4S/T5A* mutant are observed in strains deleted for *BUR2* (Figure 3B). This suggests that the mechanism by which the Ccr4-Not and Bur1/2p complexes regulate H3K4 tri-methylation levels is independent of Not4p phosphorylation.

### Phosphorylation of Not4p is required for cellular stress tolerance

**Figure 4:**
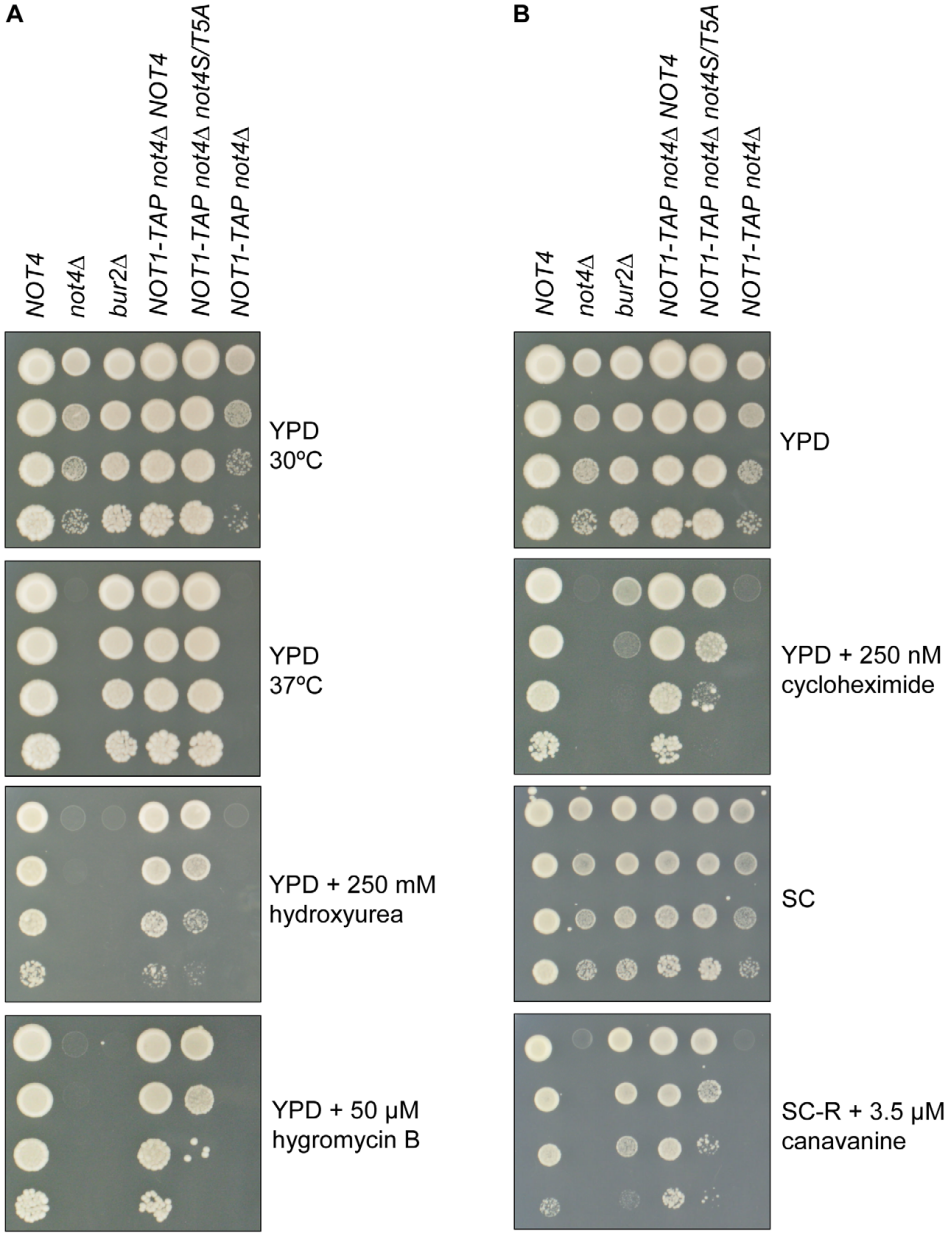
The *not4S/T5A* phenotypic analyses show overlapping drug sensitivity with the *BUR2* deletion strain. **A, B:** Strains indicated on the top were spotted in 10-fold serial dilutions on the indicated plates and incubated at 30°C (or 37°C when indicated).

The drug hydroxyurea introduces DNA replication stress [4] and yeast strains deleted for *NOT4* or *BUR2* display a similar sensitivity to hydroxyurea, which is supported by a reduced induction of *RNR3* mRNA upon hydroxyurea treatment (Figure S1). Besides this drug, yeast *NOT4* deletion mutants are also sensitive to high temperature and hygromycin B, which leads to errors during protein synthesis [6]. To explore the role of Not4p phosphorylation under these stress conditions, the *not4S/T5A* strain was subjected to 37°C, hydroxyurea or hygromycin B growth conditions. The *not4S/T5A* strain shows a temperature tolerance at 37°C unlike the *NOT4* deletion strain (Figure 4A). Both *not4Δ* and *bur2Δ* mutants are highly sensitive to hydroxyurea and hygromycin B, while the *not4S/T5A* yeast strain is weakly sensitive to hydroxyurea and mildly sensitive to hygromycin B (Figure 4A). To further examine the role of phosphorylated Not4p in the protein processing pathways, *not4S/T5A* mutants were tested for sensitivity to cycloheximide, an inhibitor for protein synthesis, and to canavanine, an arginine analog that induces protein misfolding. The *not4S/T5A* strain displays a similar sensitivity to cycloheximide and canavanine as the *bur2Δ* strain, while the growth of *not4Δ* strains is severely reduced under these conditions (Figure 4B). Notably, different combinations of Not4p phospho-site mutations resulted in wild-type growth on the indicated drug plates (Figure S2). These results indicate a redundancy among the five serine/threonine sites on Not4p. Moreover, phosphorylation of these sites is functionally important, but not essential, for resistance to replication stress and for proper processing of proteins in the cell.

**Figure 5:**
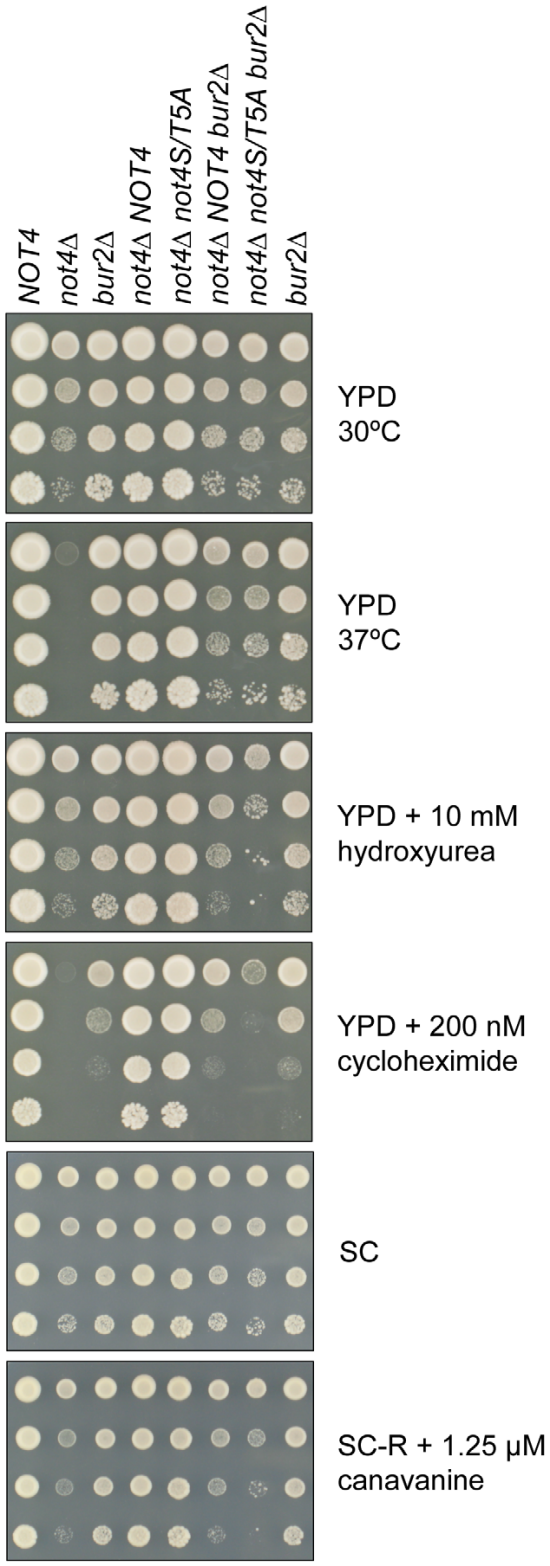
Yeast strains containing both *not4S/T5A* and *BUR2* deletion show increased drug sensitivity. Strains indicated on the top were spotted in 10-fold serial dilutions on the indicated plates and incubated at 30°C (or 37°C when indicated). Last three lanes of each plate show yeast strains with an integration of *URA3* to inactivate the *BUR2* locus.

The drug sensitivity assays showed overlapping effects for phosphorylated Not4p and Bur2p (Figure 4). To explore the synthetic genetic relationship between *BUR2* and phosphorylation of Not4p, the *not4S/T5A* and *bur2Δ* double mutant was assayed for drug tolerance levels. Interestingly, this double mutant is more sensitive for hydroxyurea, cycloheximide and canavanine than its single mutants (Figure 5). Taken together, the observed additional effect of the combination of *not4pS/T5A* and *BUR2* deletion suggests that (phosphorylation of) Ccr4-Not and Bur1/2p complexes function in parallel molecular pathways to resist DNA replication stress and cellular stress upon misfolded and/or mistranslated proteins.

## Discussion

In this study, we describe that Not4p is a phospho-protein *in vivo* (Figures 1A and 1B) and that this protein modification is not dependent on the kinase activity of Bur1p (Figure 1C). Our mass spectrometry analyses confirmed several phosphorylation sites on Not4p (S92, S312, S542/T543, putatively T334 and/or S342), Not1p (T2102) and Caf1p (S39) (Figure 1A). Absence of Not4p phosphorylation preserves the Ccr4-Not complex stoichiometry and H3K4 tri-methylation levels (Figures 2A and 3), but results in sensitivity to drugs that induce replication stress or aberrant protein synthesis (Figure 4). Moreover, the combination of a *BUR2* deletion with the Not4p phospho-pentamutation leads to a more severe phenotype than single phospho-mutants (Figure 5), indicating that a synthetic genetic relationship between phosphorylated Not4p and *BUR2* exist for various cellular stresses.

The identification of Not4p phospho-acceptor sites on SP/TP positions (Figure 1A) suggests that Not4p is a substrate for CDK/cyclin kinase pairs. The replacement of *BUR1* by the *bur1-23* allele resulted in a severely reduced Bur1p kinase activity [10], but had no effect on the phosphorylation status of Not4p (Figure 1C). This suggests that Not4p is not a direct substrate for Bur1/2 kinase activity, and other CDK/cyclin-kinase complexes may be required for Not4p phosphorylation. Ctk1p is, like Bur1p, a cyclin-dependent kinase that associates with the transcription elongation complex. It is suggested that Ctk1p and Bur1p are paralogues of the higher eukaryotic Cdk9 protein based on their sequence similarities [10]. Mutants deleted for *CTK1* did not alter the electrophoretic mobility of Not4p (data not shown), indicating that this CDK is not the required kinase for Not4p. Interestingly, yeast mutants deleted for *PHO85* showed an increased electrophoretic mobility of Not4p (data not shown), suggesting that Pho85p is involved in Not4p phosphorylation. Pho85p is a CDK that interacts with ten different cyclin partners to exert its diverse roles in the regulation of cellular responses to nutrient levels, environmental conditions and progression through the cell cycle [23]. One can speculate that Not4p is a direct substrate for Pho85p or alternatively be phosphorylated by one or multiple CDK/cyclin pairs that are targets of Pho85p.

We observed that mutation of the identified phospho-acceptor sites of Not4p to alanine does not affect global H3K4 tri-methylation levels (Figure 3), indicating that phosphorylation of Not4p on S92, S312, T543, T334 and S342 does not contribute to the regulation of histone methylation. It is important to note that we achieved 87% coverage of Not4p peptides in our mass spectrometry analyses. Conceivably, Not4p could be phosphorylated at other sites not included in our analyses, but the unchanged electrophoretic mobility of Not4p penta-phosphomutant upon phosphatase treatment suggests that we covered the major phosphorylation sites on Not4p (Figure 2B). Moreover, the penta-phosphomutant is less sensitive to certain drugs compared to a *NOT4* deletion (Figure 4) and more sensitive than the different combinations of Not4p phospho-site mutants (Figure S2), suggesting that abolishment of the majority of Not4p phospho-sites and not a particular phospho-site *per se* disrupts the function of Not4p. It is formally possible that the penta-phosphomutant is defective for reasons other than removal of phospho-sites such as misfolding, which may lead to aberrant or abolished protein interactions. However, the intact Ccr4-Not complex stoichiometry in the presence of the Not4p penta-phosphomutant (Figure 2A) indicates that the stability of the Ccr4-Not complex is preserved for Not4p penta-phosphomutant to function. Another interesting point is that the enzymatic E3 ligase function of Not4p, like Not4p phosphorylation, is important during cellular stress situations. The Ubc4/5p-interaction defective and ubiquitination-inactive Not4p-L35A mutant displayed slow growth on hydroxyurea, hygromycin B and cycloheximide plates ([6]; data not shown). Moreover, the E2 Ubc4p is important for proper Not4p functioning under these conditions, since absence of Ubc4p resulted in higher sensitivity to the same drugs as seen for the Not4p-L35A mutant ([6], [24]; data not shown). In addition, the Not4p-L35A mutant, like for Not4p penta-phosphomutant, has normal levels of H3K4me3 [7]. These observations raise the possibility that Not4p phosphorylation is involved in the E3 ligase function of Not4p. Given the reduced sensitivity of the Not4 penta-phosphomutant compared to the Not4p-L35A mutant, phosphorylation of Not4p would be modulating rather than being essential for its enzymatic function.

Previous data indicated that Not4p is functionally related to Bur1/2p for global H3K4me3 in yeast, and that *NOT4* did not influence the recruitment of Bur1p or Bur2p to genetic loci [7]. These and other observations suggest that Not4p functions downstream of the Bur1/2p complex in the histone tri-methylation pathway [13], [14]. Recently, it was suggested that loss of E3 ligase activity by deleting the RING of Not4p results in elevated levels Jhd2p, the H3K4-specific demethylase in yeast [17]. This would link degradation of Jhd2p to Not4p-mediated regulation of H3K4me3. However, the L35A mutant in the RING of Not4p abolishes its E3 ligase activity, but normal H3K4me3 levels are maintained in *not4L35A* cells [7]. Results reported in this study now indicate that Not4p and Bur1/2p act in parallel pathways. First, Not4p is not a direct substrate for the kinase activity of Bur1p (Figure 1C). Secondly, *bur2Δ* strains displayed normal growth at 37°C unlike *not4Δ* strains (Figures 4A and 5). Thirdly, *bur2Δ* and *not4Δ* strains displayed different sensitivities to inhibitors of protein synthesis/folding (cycloheximide, canavanine; Figures 4B and 5). Finally, the combination of the *not4S/T5A* and *bur2Δ* alleles showed synthetic rather than epistatic growth phenotypes (Figure 5). These observations are consistent with a model wherein the Ccr4-Not and Bur1/2 kinase complexes act in parallel pathways to regulate cellular stress responses.

## Materials and Methods

### Yeast strains, genetic manipulation and plasmids

Yeast strains used in this study are listed in Table 1. TAP-tagged strains were constructed by PCR-mediated introduction of the tag to the 3′-end of the gene. The proper strains were identified by immunoblot and co-immunoprecipitation analyses. In addition, strains were tested for known phenotypes to exclude functional interference by the TAP-tag. YSB787 (*bur1Δ*) contained the *BUR1* allele on a pRS316-*URA3* marked plasmid [10]. The *NOT4* gene in this strain was TAP-tagged, followed by a transformation with pRS315-*BUR1-HA3* or pRS315-*bur1-23-HA3* [10], and subsequently selected on 0.1% 5-Fluoroorotic acid (5-FOA) plates to remove *pRS316-BUR1*. The *not4:KanMX6* and *bur2:URA3* strains were generated using a PCR fragment from genomic DNA of strain KMY58 and KMY187, respectively. Genomic *NOT4* or *not4S/T5A* mutants were obtained by integrating the pRS305-*NOT4* or pRS305-*not4S/T5A* into the *NOT4* locus in a *not4Δ* background using the SmaI restriction site in the *NOT4* promoter region (nt -226 relative to the ATG). Integrated mutants and gene disruption were verified by PCR and/or phenotypic rescue. For yeast strains and mRNA analysis used in the Supplementary material please see the Materials and Methods S1.

### Affinity purification and mass spectrometry

TAP-tag mediated protein purifications were performed essentially as described [7]. The Ccr4-Not complex was isolated from a Caf40-TAP strain and a fraction of the purified proteins was precipitated as described [25] and resolved on a 4–12% SDS-PAGE gradient gel (NuPage, Invitrogen), stained with Biosafe (Bio-Rad) and processed for mass spectrometry analyses. In-gel proteolytic digestion of Coomassie-stained bands was performed essentially as described [26], using trypsin (Roche) or trypsin/V8 (Roche). Samples were subjected to nanoflow liquid chromatography (LC, Agilent 1100 series) and concentrated on a C18 precolumn (100 µm ID, 2 cm). Peptides were separated on an analytical column (75 µM ID, 20 cm) at a flow rate of 200 nl/min and a 60 min linear acetonitrile gradient from 0 to 80%. The LC system was directly coupled to a QTOF Micro tandem mass spectrometer (Micromass Waters, UK). A survey scan was performed from 400–1200 amu s^−1^ and precursor ions were sequenced in MS/MS mode at a threshold of 150 counts. Additional analyses were performed by nanoLC-LTQ-Orbitrap-MS (Thermo). Data were processed and subjected to database searches using Proteinlynx Global Server version 2.1 (Micromass) or the MASCOT software (Matrixscience) against SWISSPROT and the NCBI nonredundant database, with a 0.25 Da mass tolerance for both precursor ion and fragment ion. The identified phospho-peptides were confirmed by manual interpretation of the spectra.

### *In vitro* dephosphorylation assays

Proteins of TAP-tagged Notp strains were captured on IgG beads and washed three times with E-buffer (20 mM HEPES-KOH pH 8, 350 mM NaCl, 10% glycerol, 0.1% Tween-20). Immunoprecipitated material was (mock-) treated at 37°C for 45 min with shrimp alkaline phosphatase (SAP) or SAP pre-incubated with 4 mM Na-Vanadate and 800 mM NaF. Alternatively, TAP-tagged purification of proteins from *NOT4* or *not4S/T5A* strains occurs via Not1-TAP. A fraction of these purified proteins were resolved on a 4–12% SDS-PAGE gradient gel and silver stained. Other fractions were incubated at 37°C for 45 min with or without SAP. All reactions were quenched by addition of 2x sample buffer and incubated at 95°C for 5 min. Proteins were subjected to immunoblot analyses.

### Western blot and antibodies

TAP-tagged proteins were detected using the antibody against the protein A moiety of the TAP-tag (PAP; Sigma). Rabbit polyclonal antibody against Not1p and Not4p was generously provided by Dr. M.A. Collart. TBP antiserum was a kind gift from Dr. P.A. Weil. For detection of H3K4 methylation status, yeast were grown in YPD and extracts were prepared as described previously [27]. Proteins were separated by 15% SDS-PAGE gels and analyzed by immunoblotting. Antibodies against H3K4me3 (Ab8580), H3K4me2 (Ab7766), H3K4me1 (Ab8895) and H3 (Ab1791) were obtained from AbCam.

### Drug sensitivity assay

Ten-fold serial dilutions of the indicated strains were spotted on YPD plates without or with the indicated concentrations of hydroxyurea, hygromycin B or cycloheximide. The indicated strains were also 10-fold serial diluted and spotted on SC plates or SC-R plates containing the indicated concentration of canavanine. The plates were grown at 30°C (or 37°C for YPD) for 3 days.

Materials and Methods S1 and Table S1 are available online.

## Supporting Information

Figure S1 Deletion of NOT4 or BUR2 leads to similar hydroxyurea sensitivity. A: Hydroxyurea (HU) sensitivity of cells lacking NOT4 or BUR2. BY4741, *not4Δ* and *bur2Δ* strains were spotted in 10-fold serial dilutions on YPD or YPD containing 25 mM or 50 mM HU. B: HU-induced RNR3 transcription in cells lacking NOT4 or BUR2. Exponentially growing BY4741, *not4Δ* and *bur2Δ* strains were treated with 200 mM HU for 2 hours in YPD. RNA was extracted and subjected to quantitative reverse-transcriptase PCR. Standard deviations of four experiments are indicated as error bars.
(0.47 MB TIF)

Figure S2 All five phospho-sites on Not4p are required for drug tolerance. Yeast strains of several combinations of Not4p phospho-site mutations were spotted in 10-fold serial dilutions on YPD or YPD containing the indicated concentrations of hygromycin B or cycloheximide. Strains were also spotted in 10-fold serial dilutions on SC or SC without arginine (R) containing the indicated concentration of canavanine. Lane 1-11 show the yeast strain NYC1 (NOT1-TAP *not3Δ*) with an integration of not4S/TxA (x  =  1, 2, 3 or 4; mutant NOT4) at the NOT4 locus. Lane 12 shows yeast strain NYC1 with wild-type NOT4 at the NOT4 locus (see Table S1 for yeast strains).
(2.57 MB TIF)

Materials and Methods S1 (0.03 MB DOC)

Table S1 (0.04 MB DOC)

## References

[1] Collart MA, Timmers HT (2004). The eukaryotic Ccr4-not complex: a regulatory platform integrating mRNA metabolism with cellular signaling pathways?. Prog Nucleic Acid Res Mol Biol.

[2] Denis CL, Chen J (2003). The CCR4-NOT complex plays diverse roles in mRNA metabolism.. Prog Nucleic Acid Res Mol Biol.

[3] Liu HY, Badarinarayana V, Audino DC, Rappsilber J, Mann M (1998). The NOT proteins are part of the CCR4 transcriptional complex and affect gene expression both positively and negatively.. EMBO J.

[4] Mulder KW, Winkler GS, Timmers HT (2005). DNA damage and replication stress induced transcription of RNR genes is dependent on the Ccr4-Not complex.. Nucleic Acids Res.

[5] Tucker M, Staples RR, Valencia-Sanchez MA, Muhlrad D, Parker R (2002). Ccr4p is the catalytic subunit of a Ccr4p/Pop2p/Notp mRNA deadenylase complex in Saccharomyces cerevisiae.. EMBO J.

[6] Mulder KW, Inagaki A, Cameroni E, Mousson F, Winkler GS (2007a). Modulation of Ubc4p/Ubc5p-mediated stress responses by the RING-finger-dependent ubiquitin-protein ligase Not4p in Saccharomyces cerevisiae.. Genetics.

[7] Mulder KW, Brenkman AB, Inagaki A, van den Broek NJ, Timmers HT (2007b). Regulation of histone H3K4 tri-methylation and PAF complex recruitment by the Ccr4-Not complex.. Nucleic Acids Res.

[8] Prelich G, Winston F (1993). Mutations that suppress the deletion of an upstream activating sequence in yeast: involvement of a protein kinase and histone H3 in repressing transcription in vivo.. Genetics.

[9] Yao S, Neiman A, Prelich G (2000). BUR1 and BUR2 encode a divergent cyclin-dependent kinase-cyclin complex important for transcription in vivo.. Mol Cell Biol.

[10] Keogh MC, Podolny V, Buratowski S (2003). Bur1 kinase is required for efficient transcription elongation by RNA polymerase II.. Mol Cell Biol.

[11] Murray S, Udupa R, Yao S, Hartzog G, Prelich G (2001). Phosphorylation of the RNA polymerase II carboxy-terminal domain by the Bur1 cyclin-dependent kinase.. Mol Cell Biol.

[12] Zhou K, Kuo WH, Fillingham J, Greenblatt JF (2009). Control of transcriptional elongation and cotranscriptional histone modification by the yeast BUR kinase substrate Spt5.. Proc Natl Acad Sci U S A.

[13] Wood A, Schneider J, Dover J, Johnston M, Shilatifard A (2005). The Bur1/Bur2 complex is required for histone H2B monoubiquitination by Rad6/Bre1 and histone methylation by COMPASS.. Mol Cell.

[14] Laribee RN, Krogan NJ, Xiao T, Shibata Y, Hughes TR (2005). BUR kinase selectively regulates H3 K4 trimethylation and H2B ubiquitylation through recruitment of the PAF elongation complex.. Curr Biol.

[15] Pokholok DK, Harbison CT, Levine S, Cole M, Hannett NM (2005). Genome-wide map of nucleosome acetylation and methylation in yeast.. Cell.

[16] Laribee RN, Shibata Y, Mersman DP, Collins SR, Kemmeren P (2007). CCR4/NOT complex associates with the proteasome and regulates histone methylation.. Proc Natl Acad Sci U S A.

[17] Mersman DP, Du HN, Fingerman IM, South PF, Briggs SD (2009). Polyubiquitination of the demethylase Jhd2 controls histone methylation and gene expression.. Genes Dev.

[18] Albuquerque CP, Smolka MB, Payne SH, Bafna V, Eng J (2008). A multidimensional chromatography technology for in-depth phosphoproteome analysis.. Mol Cell Proteomics.

[19] Gnad F, de Godoy LM, Cox J, Neuhauser N, Ren S (2009). High-accuracy identification and bioinformatic analysis of in vivo protein phosphorylation sites in yeast.. Proteomics.

[20] Gruhler A, Olsen JV, Mohammed S, Mortensen P, Faergeman NJ (2005). Quantitative phosphoproteomics applied to the yeast pheromone signaling pathway.. Mol Cell Proteomics.

[21] Roguev A, Schaft D, Shevchenko A, Pijnappel WW, Wilm M (2001). The Saccharomyces cerevisiae Set1 complex includes an Ash2 homologue and methylates histone 3 lysine 4.. EMBO J.

[22] Dehe PM, Dichtl B, Schaft D, Roguev A, Pamblanco M (2006). Protein interactions within the Set1 complex and their roles in the regulation of histone 3 lysine 4 methylation.. J Biol Chem.

[23] Huang D, Friesen H, Andrews B (2007). Pho85, a multifunctional cyclin-dependent protein kinase in budding yeast.. Mol Microbiol.

[24] Chuang SM, Madura K (2005). Saccharomyces cerevisiae Ub-conjugating enzyme Ubc4 binds the proteasome in the presence of translationally damaged proteins.. Genetics.

[25] Wessel D, Flugge UI (1984). A method for the quantitative recovery of protein in dilute solution in the presence of detergents and lipids.. Anal Biochem.

[26] Kinter M, Sherman NE (2000). Protein Sequencing Identification Using Tandem Mass Spectrometry..

[27] Kushnirov VV (2000). Rapid and reliable protein extraction from yeast.. Yeast.

[28] Tong AH, Evangelista M, Parsons AB, Xu H, Bader GD (2001). Systematic genetic analysis with ordered arrays of yeast deletion mutants.. Science.

