# Supplementary figures and images for: Phosphorylation of Not4p Functions Parallel to BUR2 to Regulate Resistance to Cellular Stresses in Saccharomyces cerevisiae
Source: PLoS One. 2010 Apr 8;5(4):e9864. doi: 10.1371/journal.pone.0009864 (PMC2851644; doi:10.1371/journal.pone.0009864)

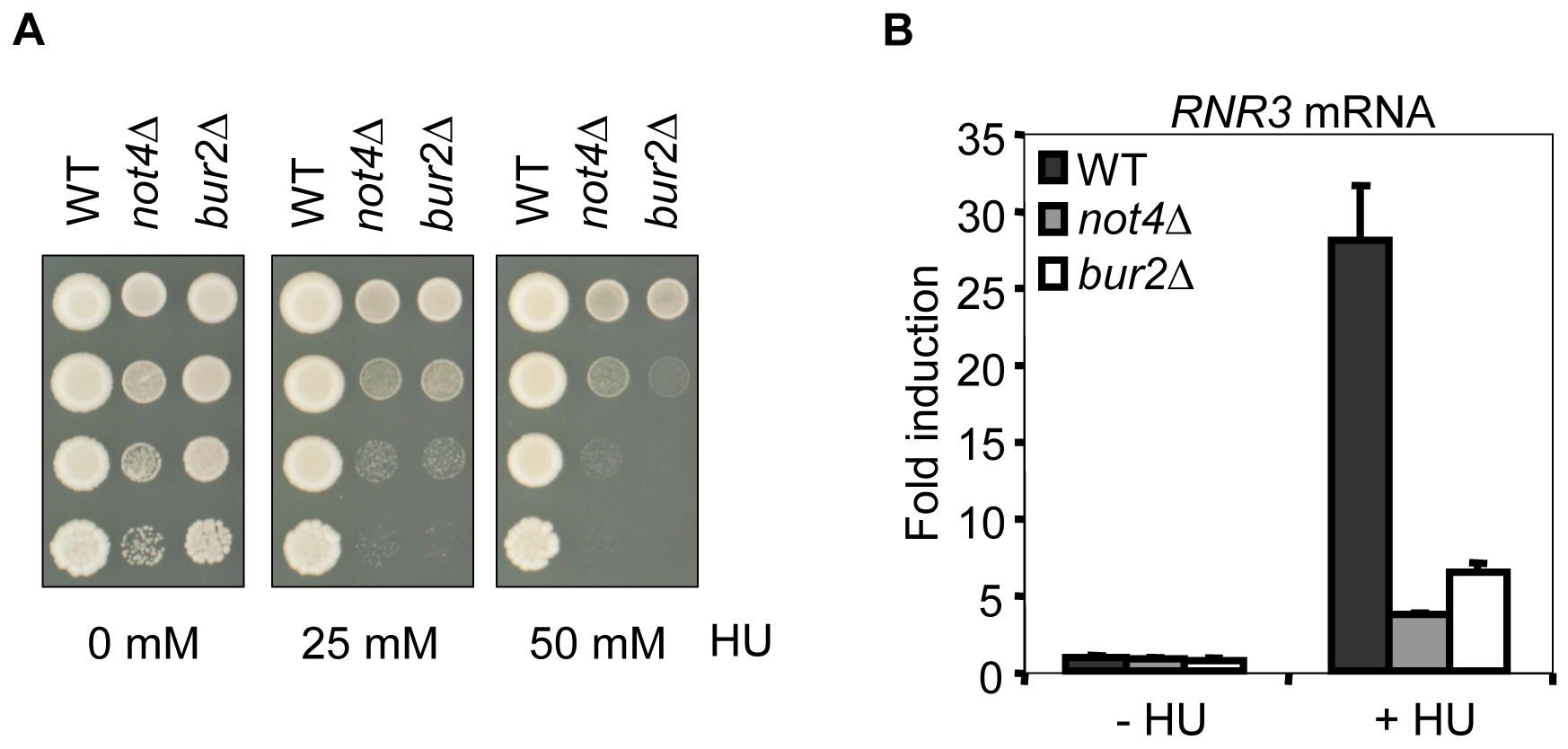

Supplement: Figure S1 — Deletion of NOT4 or BUR2 leads to similar hydroxyurea sensitivity. A: Hydroxyurea (HU) sensitivity of cells lacking NOT4 or BUR2. BY4741, not4Δ and bur2Δ strains were spotted in 10-fold serial dilutions on YPD or YPD containing 25 mM or 50 mM HU. B: HU-induced RNR3 transcription in cells lacking NOT4 or BUR2. Exponentially growing BY4741, not4Δ and bur2Δ strains were treated with 200 mM HU for 2 hours in YPD. RNA was extracted and subjected to quantitative reverse-transcriptase PCR. Standard deviations of four experiments are indicated as error bars. (0.47 MB TIF) [file pone.0009864.s001.tif]

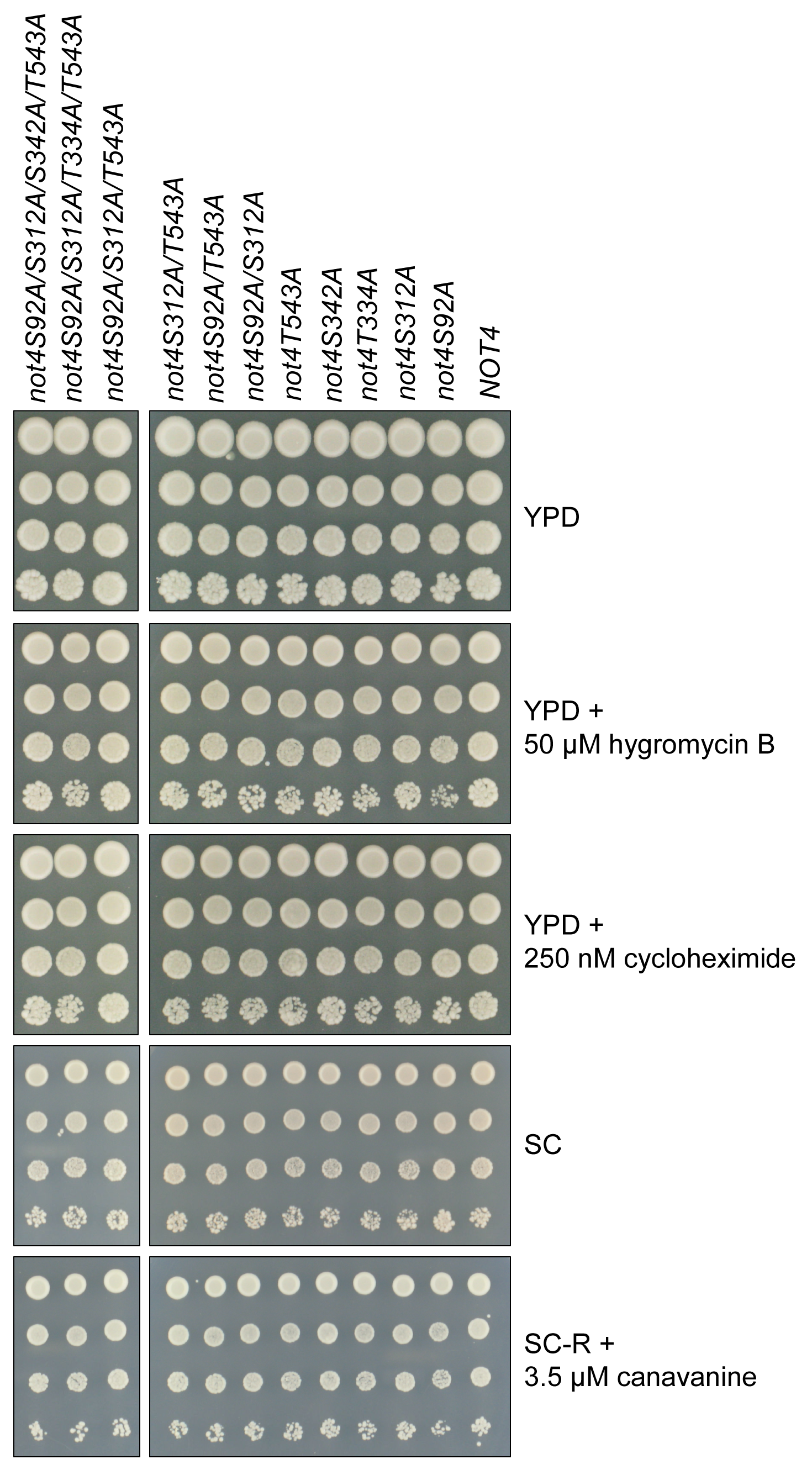

Supplement: Figure S2 — All five phospho-sites on Not4p are required for drug tolerance. Yeast strains of several combinations of Not4p phospho-site mutations were spotted in 10-fold serial dilutions on YPD or YPD containing the indicated concentrations of hygromycin B or cycloheximide. Strains were also spotted in 10-fold serial dilutions on SC or SC without arginine (R) containing the indicated concentration of canavanine. Lane 1-11 show the yeast strain NYC1 (NOT1-TAP not3Δ) with an integration of not4S/TxA (x = 1, 2, 3 or 4; mutant NOT4) at the NOT4 locus. Lane 12 shows yeast strain NYC1 with wild-type NOT4 at the NOT4 locus (see Table S1 for yeast strains). (2.57 MB TIF) [file pone.0009864.s002.tif]
