# Supplementary material for: Phosphorylation of Not4p Functions Parallel to BUR2 to Regulate Resistance to Cellular Stresses in Saccharomyces cerevisiae
Source: PLoS One. 2010 Apr 8;5(4):e9864. doi: 10.1371/journal.pone.0009864 (PMC2851644; doi:10.1371/journal.pone.0009864)
Supplement: Materials and Methods S1 — (0.03 MB DOC) [file pone.0009864.s003.doc]

## Supporting Information

**Supplementary Materials and Methods**

**Yeast strains, genetic manipulation and plasmids**

Strains used in Figures S1 and S2 are listed in Table S1. The KMY86 strain was constructed by PCR-mediated attachment of the TAP-tag to the 3’-end of the *NOT1* gene in the BY4741 background. This strain was checked by immunoblot and co-immunoprecipitation analyses. The NCY1 strain was generated via a PCR fragment from the *NOT4* locus of strain KMY58 into the KMY86 strain. The NCY1 strain was checked for temperature sensitivity at 37ºC. Genomic *NOT4* or *not4S/TxA* mutants (*x* = 1, 2, 3 or 4) were obtained by integrating the pRS305-*NOT4* or pRS305-*not4S/TxA* into the *NOT4* locus of the NCY1 strain using the SmaI restriction site in the *NOT4* promoter region (nt -226 relative to the ATG). Integrated mutants and gene disruption were verified by PCR or phenotypic analyses.

**Drug sensitivity assay**

Ten-fold serial dilutions of the indicated strains were spotted on YPD plates without or with the indicated concentrations of hydroxyurea, hygromycin B or cycloheximide. The indicated strains were also 10-fold serial diluted and spotted on SC plates or SC-R plates containing the indicated concentration of canavanine. All plates were grown at 30ºC for 3 days.

**Quantitative PCR analysis**

RNA extraction, reverse-transcription and quantitative PCR analysis were performed as described previously [Mulder, K. W., Winkler, G. S. and Timmers, H. T. (2005) DNA damage and replication stress induced transcription of RNR genes is dependent on the Ccr4-Not complex. Nucleic Acids Res **33**, 6384-92].
