# Supplementary material for: Phosphorylation of Not4p Functions Parallel to BUR2 to Regulate Resistance to Cellular Stresses in Saccharomyces cerevisiae
Source: PLoS One. 2010 Apr 8;5(4):e9864. doi: 10.1371/journal.pone.0009864 (PMC2851644; doi:10.1371/journal.pone.0009864)
Supplement: Table S1 — (0.04 MB DOC) [file pone.0009864.s004.doc]

**Table S1.** *Saccharomyces cerevisiae* strains used in the Figures S1 and S2.

| **Strain** | **Genotype** | **Source** |
| --- | --- | --- |
| BY4741 | MATa *his3Δ1 leu2Δ0 met15Δ0 ura3Δ0* | EUROSCARF |
| KMY58 | Isogenic to BY4741 except *not4:KanMX6* | EUROSCARF |
| KMY161 | Isogenic to BY4741 except *bur2:KanMX6* | EUROSCARF |
| KMY86 | Isogenic to BY4741 except *NOT1-TAP:URA3* | This work |
| NCY1 | Isogenic to KMY86 except *not4:KanMX6* | This work |
| NCY3 | Isogenic to NCY1 except *NOT4:LEU2* | This work |
| NCY4 | Isogenic to NCY1 except *not4S92A:LEU2* | This work |
| NCY5 | Isogenic to NCY1 except *not4S312A:LEU2* | This work |
| NCY6 | Isogenic to NCY1 except *not4T543A:LEU2* | This work |
| NCY7 | Isogenic to NCY1 except *not4S342A:LEU2* | This work |
| NCY8 | Isogenic to NCY1 except *not4T334A:LEU2* | This work |
| NCY9 | Isogenic to NCY1 except *not4S92A/T543A:LEU2* | This work |
| NCY10 | Isogenic to NCY1 except *not4S92A/S312A:LEU2* | This work |
| NCY11 | Isogenic to NCY1 except *not4S312A/T543A:LEU2* | This work |
| NCY12 | Isogenic to NCY1 except *not4S92A/S312A/T543A:LEU2* | This work |
| NCY13 | Isogenic to NCY1 except *not4S92A/S312A/S342A/T543A:LEU2* | This work |
| NCY14 | Isogenic to NCY1 except *not4S92A/S312A/T334A/T543A:LEU2* | This work |
